# Loads and loads and loads: the influence of prospective load, retrospective load, and ongoing task load in prospective memory

**DOI:** 10.3389/fnhum.2015.00322

**Published:** 2015-06-02

**Authors:** Beat Meier, Thomas D. Zimmermann

**Affiliations:** Institute of Psychology and Center for Cognition, Learning and Memory, University of BernBern, Switzerland

**Keywords:** prospective memory, intention, cognitive load, cognitive resources, remembering, future thinking

## Abstract

In prospective memory tasks different kinds of load can occur. Adding a prospective memory task can impose a load on ongoing task performance. Adding ongoing task load (OTL) can affect prospective memory performance. The existence of multiple target events increases prospective load (PL) and adding complexity to the to-be-remembered action increases retrospective load (RL). In two experiments, we systematically examined the effects of these different types of load on prospective memory performance. Results showed an effect of PL on costs in the ongoing task for categorical targets (Experiment 2), but not for specific targets (Experiment 1). RL and OTL both affected remembering the retrospective component of the prospective memory task. We suggest that PL can enhance costs in the ongoing task due to additional monitoring requirements. RL and OTL seem to impact the division of resources between the ongoing task and retrieval of the retrospective component, which may affect disengagement from the ongoing task. In general, the results demonstrate that the different types of load affect prospective memory differentially.

In everyday life we often have to remember to carry out an intended action at some point in the future, such as remember to buy groceries on the way home from work. This type of memory has been labeled *prospective memory* (Einstein and McDaniel, 1990). A prospective memory task always consists of two separate components: a prospective component referring to remembering *that* something has to be done and a retrospective component referring to *what* has to be done. It is mainly the prospective component that distinguishes prospective memory tasks from retrospective memory tasks. Therefore, the retrospective component is usually kept as simple as possible in laboratory experiments. Nevertheless, it is possible that features of the retrospective component such as its cognitive load affect performance. The impact of manipulating the retrospective load (RL) on prospective memory performance is the main focus of this study.

Different kinds of load exist in prospective memory tasks. First, *prospective memory load* *(PML)* refers to the additional demands that emerge for ongoing task processing when a prospective memory task is added (West and Bowry, 2005; West et al., 2006). It is typically measured as costs in ongoing task performance (Brandimonte et al., 2001). Second, *ongoing task load* *(OTL)* refers to the demands of the task in which the prospective memory task is embedded (e.g., Einstein et al., 1997; Kidder et al., 1997; Otani et al., 1997). Third, *prospective load* *(PL)* refers to the number of potential prospective memory targets or target categories that are relevant for one or more pending prospective memory tasks (Einstein et al., 1992, 2005). Finally, *RL* can be defined as number of to be remembered actions for a given set of prospective memory targets. In this paper we first review the effects of PML, OTL, PL, and RL on prospective memory performance. Then we present two experiments which were designed to systematically investigate the effect of different types of load on prospective memory performance.

## Prospective Memory Load

The question whether PML affects ongoing task processing has been particularly influential in the debate whether prospective memory retrieval is the result of automatic or controlled processes (cf. Einstein and McDaniel, 2010; Smith, 2010). PML costs in the ongoing task may reveal that processing resources are required for prospective memory retrieval whereas the lack of costs may indicate that prospective memory retrieval occurred automatically. According to the *Preparatory attentional and memory (PAM) processes theory*, PML costs occur for every prospective memory task, because participants rely on preparatory attentional processes to notice the prospective memory targets and respond accurately (Smith, 2003; Smith and Bayen, 2006). Accordingly, slowing in ongoing task processing can be found when a prospective memory task is added compared to a baseline condition with no prospective memory task (Smith, 2003; West and Bowry, 2005; Smith and Bayen, 2006; West et al., 2006). Additionally, PML costs are related to prospective memory performance with better prospective memory performance for participants who have higher PML costs (Smith, 2003). However, in other studies PML did not affect ongoing task processing and no cost emerged from adding a prospective memory task (Brandimonte et al., 2001; Einstein et al., 2005). The latter results are consistent with the *noticing plus search model* (Einstein and McDaniel, 1996). According to this approach noticing a prospective memory target is an automatic process while retrieving the intention relies on processing resources.

According to the multiprocess view, the amount of resulting PML costs depends on the characteristics of the prospective memory task, the ongoing task and individual differences. Empirically, PML costs occurred when the importance of the prospective memory task was emphasized (Kliegel et al., 2004; Einstein et al., 2005; see Walter and Meier, 2014, for an overview), when the prospective memory task was encoded as a vigilance task (Brandimonte et al., 2001), for unrelated rather than for related cues (Marsh et al., 2003), for ill-specified rather than for well-specified intentions (Hicks et al., 2005), and under high PL (Einstein et al., 2005). In line with these results, we found higher PML costs for individuals who reported to have been successful due to a strategic *search* for the prospective memory targets in a previous study; in contrast, individuals who reported a *pop up* experience when they encountered a prospective memory target did not display costs in the ongoing task (Meier et al., 2006b).

## Ongoing Task Load

Although different types of load can occur in prospective memory tasks, most studies have focused on OTL. Different manipulations can be used to manipulate OTL. First, it can be manipulated by varying *ongoing task difficulty*. Second, it can be manipulated by adding an *additional concurrent ongoing task*. Third, it can be manipulated by varying the *difficulty of the additional ongoing task*. Depending on the manipulation, different effects have been reported.

Kidder et al. (1997) varied ongoing task difficulty to manipulate OTL. They used a verbal working memory task as an ongoing task for which participants had to recall either two or three preceding words at unpredictable intervals. Prospective memory performance was lower in the more demanding condition (i.e., three word recall) compared to the less demanding condition (i.e., two word recall). In other studies, OTL was varied by adding a concurrent ongoing task (Einstein et al., 1997; McDaniel et al., 2004). Results showed a decrease in prospective memory performance for participants in the high load condition. Finally, in some studies OTL was varied as a function of demands of the additional ongoing task. For example, different levels of articulatory suppression revealed no effect on prospective memory performance (Otani et al., 1997; Marsh and Hicks, 1998). In contrast, additional ongoing tasks with a stronger monitoring component (e.g., arithmetic task, visuo-spatial monitoring, counting and random number generation) affected prospective memory performance (Marsh and Hicks, 1998; Logie et al., 2004).

Because these studies did not assess the prospective and retrospective components of prospective memory separately it remains unclear which component is more affected by OTL. According to the PAM theory noticing the prospective memory cue is the consequence of preparatory attentional processes and therefore OTL may affect mainly the prospective component. Specifically, high OTL may limit the available processing resources to strategically search for prospective memory cues or to maintain a state of readiness to perform the prospective memory task. However, OTL may also affect the retrospective component, in particular the ease with which participants disengage from the ongoing task and switch to the prospective memory task. For example, when OTL is manipulated by increasing the pace of the ongoing task it is more difficult to disengage and shift away from the dominant mental set of the ongoing task (Graf, 2005).

More recently, a few studies have assessed the prospective and retrospective components of prospective memory separately using a multinomial modeling approach. Horn et al. (2011) found that manipulations of ongoing-task difficulty affected the ongoing task parameters of the model, leaving the estimates for the prospective and the retrospective components unaffected. In a study with younger and older adults, Smith et al. (2012) adjusted ongoing task difficulty and still found lower prospective memory performance for older compared to younger adults, in particular for the prospective component. In a follow-up study, Smith and Hunt (2014) found that this pattern of results persisted independent of whether the instructions emphasized the prospective or the ongoing task. Thus, these studies suggest that OTL primarily affects ongoing task performance but does not differentially influence the model parameters of the prospective and the retrospective component in a multinomial model.

## Prospective Load

In order to investigate PL, Einstein et al. (1992) varied the number of different prospective memory targets. In one condition participants had to respond to a specific target word (e.g., rake), in the other condition they had to respond to four different target words (rake, truck, nose, and soap). In both conditions three prospective memory targets occurred in the ongoing short-term memory task. The results showed higher prospective memory performance for the one-word condition than for the four-word condition. In addition, the effect of PL was stronger for older adults than for younger adults. A similar finding was reported by Kidder et al. (1997). They found an effect of PL for older but not for younger adults. Similarly, in a study by Einstein et al. (2005), PL did not affect prospective memory performance. However, there were PML costs in the ongoing task with slowing for the six-target condition, but not for the one-target condition. Einstein and colleagues contemplated that under high PL participants may have rehearsed the target events more often and/or may have allocated more resources to strategic monitoring for the target events.

Marsh et al. (2003) also manipulated the number of prospective memory targets (four vs. eight). In addition, they also varied the semantic relatedness of the targets. None of these manipulations affected prospective memory performance. However, Marsh et al. (2003) found PML costs for the conditions with unrelated targets. They suggested that for related target events performance facilitation may have occurred as a result of categorical priming.

Cohen et al. (2008) systematically manipulated the number of prospective memory targets from 1 to 6. With 1 or 2 targets, performance in the ongoing lexical decision task was not significantly affected. However, with more targets significant costs occurred. Specifically, there was a linear relationship between the number of targets and slowing for the word trials of the ongoing task but not for the non-word trials.

Importantly, in all these studies target events were repeated in the low PL condition. Therefore, the performance advantage in this condition may have materialized at least in part as a result of processing facilitation due to target repetition. To circumvent this problem, Einstein et al. (1992) compared performance of the first target only, and this additional analysis did not show a statistically significant effect of PL. In this study, we used a different approach. PL was manipulated by using prospective memory targets from one vs. four taxonomic categories. As a consequence target repetition was not necessary for the low PL condition and the number of prospective memory targets was constant across conditions.

## Retrospective Load

So far, no study has directly examined the effect of RL on prospective memory performance. However, there are two relevant studies that primarily addressed output monitoring in prospective memory (Marsh et al., 2002, 2007). In these studies, the same prospective memory targets were presented repeatedly, and participants (younger and older adults) were instructed to press a different response key when a target re-appeared. The results showed that younger adults were less likely to forget successful responses. They were more likely to falsely claim that they had responded to an earlier target. In contrast, older adults were more likely to forget they had already responded to a particular target (Marsh et al., 2002). Because this study was designed to examine output monitoring effects it did not include a baseline condition, that is, a condition for which participants had to respond to all targets with the same key-press. Nevertheless, it is possible that the number of to-be-remembered actions affected RL. In the present study, we used a similar procedure to manipulate RL. In the low RL condition participants had to press the same specific key for every target event. In the high RL load condition, participants had to press a different pre-specified key every time a target event appeared. By design, this condition also involved an output monitoring component because participants had to remember how many targets they had already responded to.

## Overview of the Present Study

To date, no study has systematically analyzed the effects of PML, OTL, PL, and RL. The present work was designed to fill this gap. The impact of different types of load was measured separately for the prospective component and the retrospective component. Our method is based on typical behavior patterns of participants in prospective memory experiments. Upon recognition of a prospective memory target participants often move back in their chairs, sometimes accompanied by exclamations like “oops”, “aha”, “now I have to do something”, “what I am supposed to do now?” (cf. Meier et al., 2006b, 2011). At this point, the prospective component (remember that) is fulfilled, but participants do not necessarily know yet what exactly they have to do, that is, the retrospective component (remember what) still has to be remembered. These observations are consistent with the noticing plus search model, in which processes related to detecting target events and processes related to retrieving the contents of the intention are differentiated (Einstein and McDaniel, 1996; cf. Rothen and Meier, 2014). In order to experimentally distinguish the prospective and retrospective component, participants were required to continuously press a specific key (i.e., the shift-key) during the ongoing task. The prospective memory task was to press a different key with the same finger as soon as a prospective memory target event occurred. To fulfill this request, participants had to release the shift-key by design. Therefore, releasing the shift-key is an indicator of the prospective component and pressing the appropriate key is an indicator of the retrospective component.

In conventional prospective memory tasks the prospective and retrospective components are not measured separately. Typically, the retrospective component is kept as simple as possible (i.e., pressing a key) to allow for a pure measurement of the prospective component. Nevertheless, it is still possible that participants detect a prospective memory target and correctly interrupt the ongoing task, but then fail to retrieve the retrospective component. If this happens, the prospective memory measure is contaminated by retrospective memory failure. In previous studies we have shown that this type of error is more likely to occur in older adults (Zimmermann and Meier, 2006, 2010). It is widely assumed that aging reduces the capacity of available processing resources. Because enhancing cognitive load also reduces available processing resources in a prospective memory task one might expect similar results for younger adults under high load conditions as for older adults.

In the following two experiments, we orthogonally manipulated load of the prospective and retrospective components. In Experiment 1, specific prospective memory targets were used, in Experiment 2, categorical targets were used. In Experiment 2, OTL was also manipulated. In both experiments we used a lexical decision task as ongoing activity. In order to keep ongoing task difficulty constant for all participants, the pace of presentation was individually adjusted.

## Experiment 1

In Experiment 1, PL was manipulated by varying the number of taxonomic categories from which the prospective memory cues were drawn. In the low PL condition prospective memory targets were four specific words from one target category (i.e., animals). In the high PL condition prospective memory targets were four specific words from four taxonomic categories (i.e., animals, vehicles, musical instruments and sports equipment). With high PL more resources may be required to detect prospective memory targets. Therefore, we expected that higher PL would increase PML costs. As target events were not presented repeatedly in the low PL condition, we did not expect an effect of PL for prospective memory performance (cf. Einstein et al., 1992, 2005; Kidder et al., 1997).

RL was manipulated by increasing the number of to-be-remembered actions. In the low RL condition, participants had to press the same specific key for each and every target event. In the high RL load condition, participants had to press a different pre-specified key every time a target event appeared (i.e., “1” for the first target, “2” for the second target, etc.). As the latter condition required that participants kept track of the number of encountered prospective memory targets, we expected RL to affect the retrospective component of prospective memory performance.

### Method

#### Participants and Design

Sixty young adults between 18 and 30 years (*M* = 22.5, *SD* = 2.98) participated in this study. They were undergraduate volunteers from the University of Bern. The orthogonal combination of PL and RL resulted in a 2 × 2 between-subjects design. Participants were randomly assigned to one of the four experimental conditions. In each condition 15 participants were tested. The study was approved by the local ethical committee of the University of Bern, and all participants gave informed consent.

#### Material and Apparatus

For the lexical decision task a total of 124 German nouns and 128 nonwords were selected. For every word a non-word was generated by changing the order of letters. One set of 16 words and 16 non-words was used to adjust ongoing task difficulty, and another set of 16 words and 16 non-words was used in the baseline task. Another set of 92 words and 96 nonwords was used in the ongoing task, complemented by an additional set of four prospective memory target words. In the low PL condition, prospective memory targets were selected from one category (animals) and consisted of the German nouns “Pferd” (horse), “Vogel” (bird), “Fuchs” (fox), and “Frosch” (frog). In the high PL condition, prospective memory targets were selected from each of the four categories “animals”, “vehicles”, “musical instruments” and “sports equipment”. Prospective memory targets were “Pferd” (horse), “Auto” (car), “Geige” (violin), and “Ball” (ball). Word frequencies did not differ for the two sets of prospective memory targets, *t*_(6)_ = 1.19, *p* > 0.28 (CELEX 2 database, Baayen et al., 1995). Words and nonwords were presented in 18-point font at the center of a 15” VGA-monitor. Presentation of stimuli was controlled by E-Prime 1.1 software (Psychology Software Tools)^1^ running on IBM-compatible computers.

#### Procedure

**Figure 1:**
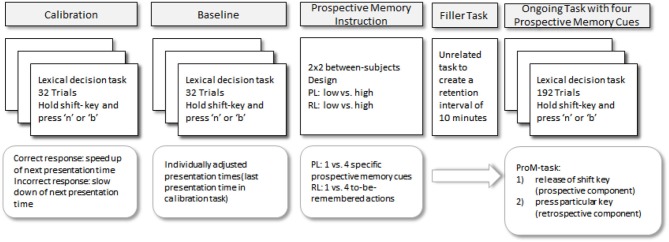
**Schematic depiction of the procedure for Experiment 1**.

The procedure is depicted in Figure 1. After giving consent, participants were instructed on the lexical decision task. They were told that they would be presented with letter-strings and that for each string they had to make a word/nonword-decision by pressing the appropriate key with the index finger and the middle finger of the right hand (i.e., the “b” and “n”-keys) as fast and as accurately as possible. They were also instructed to continuously press the shift-key with the left index finger to keep the task going. They were told that if they mistakenly released this key, the program would stop until they pressed it again. These instructions were explained until participants understood and were able to repeat them. Next, 32 trials of the lexical decision task were administered in order to individually adjust the level of difficulty for each individual. Specifically, presentation time of the stimuli was adjusted for the baseline and the ongoing task. Stimuli were presented in pseudo-randomized order with an initial presentation time of two seconds. Each trial started with a black fixation cross at the center of the screen for 1 s, followed by a 250 ms blank screen, which preceded the presentation of the stimulus. As soon as participants responded the next trial started. Every time a correct answer was given, the presentation time for the next trial decreased by 125 ms (with a lower bound of 125 ms). Every time an incorrect answer was given, presentation time increased by 125 ms (no upper bound was set). The presentation time of the last trial was used to individually adjust the presentation time for each participant for the rest of the experiment. Mean presentation time was 279 ms (*SD* = 62 ms). Specifically, for 48 participants presentation time was 250 ms, for 10 participants it was 375 ms, and for two it was 500 ms. An analysis of variance (ANOVA) with the four different groups as between-subject factor revealed no significant differences (*F*_(3,57)_ = 1.04, *p* > 0.37). Next, the baseline phase followed, in which a total of 16 words and 16 nonwords were presented. Stimuli were presented for 120% of the individually adjusted presentation time.

Then, participants were instructed on the prospective memory task. They were informed that an additional goal of the study was to investigate how well they could remember to carry out an intended activity in the future. For the low RL groups the activity was to press a particular key (“1”) on the keyboard for every target. The high RL groups were requested to press a different key for each prospective memory target (“1” for the first detected prospective memory target, “2” for the second one and so on). Specifically, they were instructed to press the appropriate key on the keyboard with the left index finger whenever they saw a prospective memory target. Moreover, for half of each RL group, the low PL condition, the prospective memory targets were four specific animal words (i.e., horse, bird, fox, and frog), and for the other half, the high PL condition, prospective memory targets were four specific words from the categories “animals”, “vehicles”, “musical instruments” and “sports equipment” (i.e., horse, car, violin, and ball). The instructions were explained until participants understood and were able to repeat them.

Next, an unrelated questionnaire was administered for 10 min to create a filled retention interval. Then, the ongoing task containing the prospective memory targets was started. The prospective memory task was not mentioned again. A total of 192 trials were presented. Prospective memory targets occurred on the 47th, 95th, 143rd, and 191st trial. The selection of one of four prospective memory targets was random without replacement. Whenever participants released the shift-key, the ongoing task was interrupted. When they appropriately released the shift-key and pressed any of the four keys for the prospective memory task, a screen with the request to “press the shift-key to continue” appeared.

At the end of the experiment, participants who failed to correctly respond to any of the four prospective memory targets were asked whether they remembered the instructions for the prospective memory task. All participants managed to recall them accurately.

### Results

**Figure 2:**
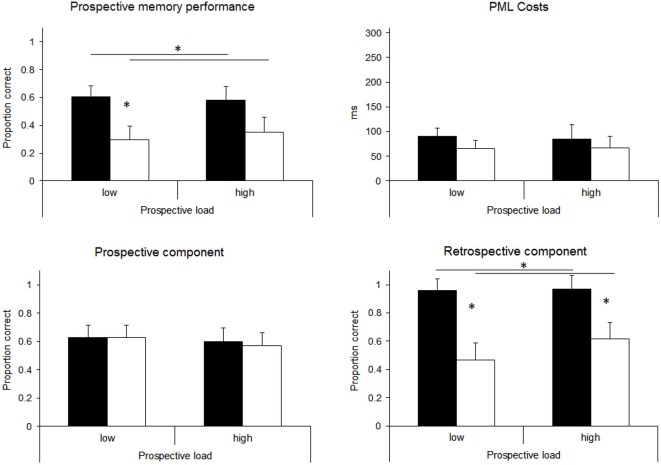
**Results of Experiment 1, separately for overall prospective memory performance, prospective memory load (PML) Costs in the ongoing task, the prospective component (releasing the shift-key), and the retrospective component (pressing the correct response)**. Black bars represent the low retrospective load (RL) condition, white bars the high RL conditions. Significant differences (*p* < 0.05) are indicated by asterisks. Error bars represent standard errors.

For all statistical analyses alpha was set at 0.05. All reported *η*^2^ are *partial η*^2^. Prospective memory performance was measured as proportion of correct responses. First, overall prospective memory performance was analyzed. A response was scored as correct, when the shift-key was released and the appropriate key was pressed on the appropriate occasion. Proportions of correct prospective memory responses are displayed in Figure 2 (top left). A two-factorial ANOVA with PL and RL as between-subject factors revealed a significant effect of RL, *F*_(1,56)_ = 6.49, *p* < 0.05, *MSe* = 0.14, *η*^2^ = 0.10 (*M* = 0.59 for low RL and *M* = 0.35 for high RL). No other effect was significant (all *F*_s_ < 1; all *p*s > 0.5; *MSe* = 0.14). Directed comparisons revealed that the main effect of RL was mainly driven by the difference in the low PL condition, *t*_(28)_ = 2.37, *p* > 0.05, while the effect in the high PL condition was not statistically significant, *t*_(28)_ = 1.23, *p* = 0.21.

Next, prospective memory performance was analyzed separately for the prospective component and the retrospective component. For the prospective component, a response was scored as correct when the shift-key was released on the appropriate occasion. Proportions of correct responses for the prospective component are displayed in Figure 2 (bottom left). A two-factorial ANOVA with PL and RL as between-subjects factors revealed no significant main effects and no significant interaction (all *F*s < 1; all *p*s > 0.5; *MSe* = 0.12).

The retrospective component was scored as correct when the appropriate key was pressed after the shift-key had been released. For the high RL condition, a response was scored as correct, when the key-press corresponded to the correct position of individually detected prospective memory targets. Overall 54 of the 60 participants correctly interrupted the ongoing task at least once. For these participants performance on the retrospective component was calculated as conditional probabilities. Proportions of correct responses across the four groups are also shown in Figure 2 (bottom right). A two-factorial ANOVA with PL and RL as between-subject factors revealed a significant main effect of RL, *F*_(1,50)_ = 24.64, *p* < 0.01, *MSe* = 0.10; *η*^2^ = 0.33 (*M* = 0.97 for low RL and *M* = 0.54 for high RL). No other effect was significant (all *F*s < 1; all *p*s > 0.30; *MSe* = 0.10).^2^ Directed comparisons showed that correct scores on the retrospective component were higher in the low RL conditions for both low and high PL conditions, *t*_(26)_ = 3.99, *p* < 0.01 and *t*_(24)_ = 3.04, *p* < 0.01, respectively.

Reaction times for correct responses to word trials of the lexical decision task were compared for the baseline and the ongoing task. In order to exclude potential after-effects of responding to prospective memory targets, we excluded 6 trials following a prospective memory response (cf. Meier and Rey-Mermet, 2012). A paired sample *t*-test revealed that reaction times were significantly higher in the ongoing task (608 ms) than in the baseline phase (531 ms), *t*_(59)_ = 7.19; *p* < 0.01. The difference between baseline performance and ongoing task performance was calculated as a measure of PML costs. Positive values indicate a slowing in reaction times for the ongoing task compared to the baseline. Mean differences are displayed in Figure 2 (top right). A two-factorial ANOVA with PL and RL as between-subjects factors revealed no significant effects (all *F*s < 1; all *p*s > 0.25).

We also compared accuracy in the lexical decision task for baseline and ongoing task trials. A paired sample *t*-test revealed that accuracy was significantly higher in the ongoing task (92.9%) than in the baseline phase (90.6%), *t*_(59)_ = 2.37; *p* < 0.05, indicating a practice effect. A two-factorial ANOVA for the accuracy difference between baseline and ongoing task with PL and RL as between-subjects factors revealed no significant effects (all *F*s < 1; all *p*s > 0.46).

### Discussion

The goal of Experiment 1 was to examine the effect of PL and RL on prospective memory performance. The results revealed an effect of RL, but not of PL for overall prospective memory performance. Separate analyses for both components revealed that RL affected the retrospective component, but not the prospective component. Moreover, neither PL nor RL affected PML costs.

We did not find an effect of PL on prospective memory performance. This result is consistent with Einstein et al. (1992) who did not find an effect of PL when results for only the first target were considered. However, some previous studies reported PL effects when the same targets were presented repeatedly in the low PL condition. Our results suggest that target repetition resulted in an over-estimation of performance in the low PL condition in previous studies. If this facilitation is removed PL does not seem to affect performance.

In the present study, there was also no effect of PL on PML costs. This may indicate that PL does not affect performance for specific targets. However, PL effects may materialize with categorical targets. To test this possibility, categorical prospective memory targets were used in the Experiment 2.

In Experiment 1, RL affected prospective memory performance, particularly retrieval of the retrospective component. We assume that lower performance on the retrospective component in the high load condition is due to the additional requirements of updating the memory for future remembering. Because all participants were able to correctly recall the requirements for the retrospective component in the post-experimental interview, forgetting to press the correct key rather seems to be due to a transient failure of output monitoring than due to completely forgetting what one has to do. In prospective memory tasks, retrieval of the retrospective component is often resource demanding, because it also requires disengaging from the ongoing task and switching to the prospective memory task. As retrieval of the retrospective component and switching the tasks draw from the same pool of processing resources, almost simultaneously a competition between these processes occurs. As resource-demanding processes require time to complete, competition can result in a completion failure of already initiated processes (Meier et al., 2003). As a consequence, correctly performing the retrospective component can fail despite noticing the target event (i.e., the prospective component) and despite successfully retrieving the retrospective component at the end of the experiment. In a previous study, we have found that this kind of failure takes place more often in older age, that is, for individuals assumed to have reduced processing resources (Zimmermann and Meier, 2006).

In the light of these considerations, task requirements that affect disengagement and task switching are likely to affect whether retrieval of the retrospective component will be completed. Similar to old age, we assumed that the pace of the ongoing task is likely to affect the amount of available processing resources. Disengaging from a fast paced ongoing task that requires quick responses seems to be more difficult compared to disengaging from a slow paced task (cf. Meier et al., 2003). Also, competition between disengagement from the ongoing task and retrieval of the retrospective component is more accentuated for fast paced ongoing tasks. To test this assumption, OTL was manipulated in the second experiment by varying the presentation time of stimuli in the ongoing task.

## Experiment 2

Experiment 2 was identical to Experiment 1 except for the use of categorical prospective memory targets. In addition, an OTL manipulation was included.

### Method

#### Participants and Design

Hundred and fifty-three young adults between 17 and 30 years (*M* = 22, *SD* = 3.2) participated in this study. They were undergraduate volunteers from the University of Bern. PL was manipulated by using categorical prospective memory targets from either one taxonomic category (low PL) or four taxonomic categories (high PL). RL was manipulated by varying the complexity of to-be-remembered actions for the four prospective memory targets as in Experiment 1. OTL was manipulated by varying the pace of the ongoing lexical decision task. In the low OTL condition, stimuli were presented somewhat longer than the individually adjusted presentation time. In the high OTL condition, one half of the stimuli (including the four prospective memory targets) were also presented somewhat longer than the individually adjusted presentation time. The other half was presented somewhat shorter in order to increase the pace of the ongoing task. PL, RL, and OTL were crossed orthogonally, resulting in a 2 × 2 × 2 between-subjects design. Participants were randomly assigned to one of the eight experimental conditions. In each condition 18–20 participants were tested.

#### Material and Procedure

**Figure 3:**
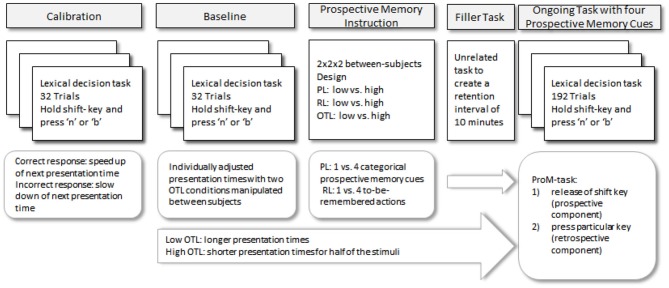
**Schematic depiction of the procedure for Experiment 2**.

The material and equipment were identical to those in Experiment 1. The procedure was almost identical and is presented in Figure 3. Here, we only highlight those aspects of the procedure that were different in Experiment 2.

In order to manipulate OTL, presentation times for the lexical decision task were varied. As in Experiment 1, 32 trials of the lexical decision task were administered to individually adjust the pace of the lexical decision task. For the low OTL condition, stimuli were presented for 120% of the individually adjusted presentation time. For the high OTL condition, half of the trials (including the prospective memory targets) were also presented for 120%. The other half of the trials were presented for 80% of the individually adjusted presentation time.

### Results

**Figure 4:**
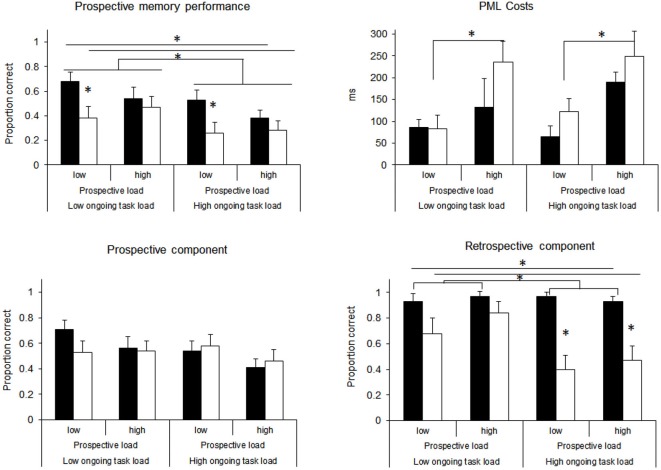
**Results of Experiment 2, separately for overall prospective memory performance, PML Costs in the ongoing task, the prospective component (releasing the shift-key), and the retrospective component (pressing the correct response)**. Black bars represent the low RL condition, white bars the high RL conditions. Significant differences (*p* < 0.05) are indicated by asterisks. Error bars represent standard errors.

First, overall prospective memory performance was analyzed. A response was scored as correct when the shift-key was released and the appropriate key was pressed on the appropriate occasion. Proportions of correct responses are displayed in Figure 4 (top left). A three-factorial ANOVA with PL, RL, and OTL as between-subject factors revealed a significant effect of RL, *F*_(1,145)_ = 9.87, *p* < 0.01, *MSe* = 0.13, *η*^2^ = 0.06, (*M* = 0.53 for low RL and *M* = 0.35 for high RL) and a significant effect of OTL, *F*_(1,145)_ = 7.32, *p* < 0.01, *MSe* = 0.13, *η*^2^ = 0.05 (*M* = 0.52 for low OTL and* M* = 0.36 for high OTL). No other effect was significant (all *F*s < 2.8; all *p*s > 0.09; *MSe* = 0.13). As the pattern of results suggests a marginal interaction between PL and RL, we conducted further analyses. For the low PL conditions, these revealed consistent significant differences between high and low RL with *t*_(36)_ = 2.50, *p* < 0.05 for the low OTL condition, and *t*_(38)_ = 2.78, *p* < 0.05 for the high OTL condition. In contrast, no significant effect materialized in the high PL conditions (*t*s < 1, *p*s > 0.33).

Next, prospective memory performance was analyzed separately for the prospective and the retrospective components. For the prospective component, a response was scored as correct when the shift-key was released on the appropriate occasion. Proportions of correct responses are displayed in Figure 4 (bottom left). A three-factorial ANOVA with PL, RL, and OTL as between-subjects factors revealed no significant effects (all *F*s < 2.5 all *p*s > 0.1; *MSe* = 0.14). Although the scores for low PL were numerically higher (*M* = 0.59) than for high PL (*M* = 0.49) this difference was not significant, *F*_(1,145)_ = 2.44, *p* = 0.12; *MSe* = 0.14.

The retrospective component was scored as correct when the appropriate key was pressed after the participants had released the shift-key. For the high RL conditions, responses were scored as correct when the response corresponded to the position of the individually detected prospective memory targets. Overall, 119 of the 153 participants correctly interrupted the ongoing task at least once. For these participants performance on the retrospective component was analyzed as a conditional probability. Proportions of correct responses across the four groups are also shown in Figure 4 (bottom right). A three-factorial ANOVA with PL, RL, and OTL as between-subject factors revealed a highly significant main effect of RL, *F*_(1,111)_ = 37.4, *p* < 0.01, *MSe* = 0.09, *η*^2^ = 0.25 (*M* = 0.95 for low RL and *M* = 0.61 for high RL), a significant main effect of OTL, *F*_(1,111)_ = 8.17, *p* < 0.01, *MSe* = 0.09, *η*^2^ = 0.07 (*M* = 0.86 for low OTL and *M* = 0.70 for high OTL) and a significant interaction between RL and OTL, *F*_(1,111)_ = 8.25, *p* < 0.01, *MSe* = 0.09, *η*^2^ = 0.07. *Post hoc* *t*-tests revealed that OTL affected performance in the high RL condition (*M* = 0.77 for low OTL and *M* = 0.43 for high OTL; *t*_(56)_ = 3.16, *p* < 0.01) but not in the low RL condition (*M* = 0.94 for low OTL and *M* = 0.95 for high OTL; *t*_(59)_ = 0.13, *p* > 0.89). No other effect was significant (all *F*s < 1.1; all *p*s > 0.3; *MSe* = 0.09).^3^

Using the same procedure in Experiment 1, we compared reaction times for correct responses to word trials of the lexical decision task for the baseline and the ongoing task. A paired sample *t*-test revealed that reaction times were significantly higher for the ongoing task (736 ms) than for the baseline (594 ms), *t*_(151)_ = 9.68, *p* < 0.01. The difference in mean reaction times for the lexical decision task between the baseline phase and the ongoing task was calculated as a measure of PML costs. Positive values indicate a slowing in reaction times for the ongoing task compared to the baseline trials. Mean differences are displayed in Figure 4 (top right). A three-factorial ANOVA with PL, RL, and OTL as between-subjects factors revealed a main effect of PL, *F*_(1,145)_ = 15.78, *p* < 0.01, *MSe* = 30491, *η*^2^ = 0.10 (*M* = 89 ms for low PL and *M* = 201 ms for high PL), and a marginally significant effect of RL, *F*_(1,145)_ = 3.63, *p* = 0.06, *MSe* = 30491, *η*^2^ = 0.02 (*M* = 118 ms for low RL and *M* = 172 ms for high RL). No other main effect and interaction reached significance (all *F*s < 1; all *p*s > 0.33).

We also compared accuracy in the lexical decision task for baseline and ongoing trials. A paired sample *t*-test revealed that accuracy was significantly higher in the ongoing task (95.0%) than in the baseline phase (92.9%), *t*_(151)_ = 3.50; *p* < 0.01, indicating a practice effect. A three-factorial ANOVA for the accuracy difference between baseline and ongoing task with PL, RL and OTL as between-subjects factors revealed no significant effects (all *F*s < 2.8; all *p*s > 0.10.

### Discussion

The goal of Experiment 2 was to replicate the RL effect of Experiment 1 and to further examine the effect of PL for categorical target events. In addition, OTL was manipulated to test whether it affects retrieval of the retrospective component. We found an effect of PL on PML costs, but not on prospective memory performance. In contrast, RL and OTL both affected prospective memory performance. Separate analyses for the prospective and the retrospective components revealed that these effects were due to failure on the retrospective component. Additionally, an interaction between these two types of load was found. High OTL affected performance on the retrospective component in the high RL condition more than in the low RL condition.

In contrast to Experiment 1, there was a nominal effect of PL on the prospective component and there was a significant PL effect on PML costs. The latter result suggests that participants attempted to compensate for higher PL by allocating more resources to the prospective memory task. Both effects are consistent with the findings of Einstein et al. (2005), who also found a nominal effect of PL on prospective memory performance and a significant effect on PML costs. According to Einstein et al. (2005) participants in the high PL condition may have rehearsed the target events more often and they may have allocated more resources for strategic monitoring. It seems plausible, that if participants use such a strategy to counter higher PL, then effects of PL on prospective memory performance—specifically the prospective component—may be minimal.

In Experiment 2, we replicated the RL effect on the retrospective component. Participants in the high RL condition were more likely to fail to retrieve the retrospective component. However, as in Experiment 1 no difference in remembering the retrospective component was found in the post-experimental interview. After the experiment all participants were perfectly able to recall the requirements for the retrospective component. It seems likely that high RL decreased the amount of available processing resources. As a consequence, a competition between processes required for disengaging from the ongoing task and processes required for retrieval of the retrospective component resulted in a lack of completion of both processes. In addition, increasing the pace of the ongoing task (i.e., increasing the difficulty for disengaging from the ongoing task) should also interact with RL. To test this assumption we manipulated OTL by varying presentation times for stimuli in the ongoing task.

Our results showed that OTL affected the retrospective component. This is consistent with our assumption. It is also in line with a proposition by Graf (2005) that shifting away from the dominant mental set of the ongoing task is more difficult when the pace of the ongoing task is high. Additionally, the interaction between RL and OTL indicates that task switching and retrieval operations relied on the same pool of processing resources. As a consequence, participants may fail to initiate the retrospective component because they cannot complete disengagement from the ongoing task or because they cannot complete retrieval of the planned action. The significant effect of RL on PML costs suggests that participants may have tried to avoid these requirements by rehearsing and maintaining the retrospective component in working memory. This strategy would slow ongoing task performance, but would reduce retrieval requirements.

## General Discussion

This study addressed the question of whether different types of load affect prospective memory performance differentially. We manipulated PL, RL, and OTL to investigate their impact on the prospective and retrospective components of prospective memory separately. Our results revealed that PL affected PML costs, RL influenced retrieving the content of the intention and performing the required action, and OTL affected the ease with which participants managed to disengage from the ongoing task. In general, load affected the retrospective component rather than the prospective component. As automatic processes are assumed to require minimal processing resources and to occur without conscious control, remembering the prospective component may be less susceptible to resource demanding load manipulation. By contrast, controlled processes are slower and resource demanding and therefore, the retrospective component is more likely to be affected by resource demanding load manipulations.

PL showed no effect for specific targets (Experiment 1) but affected PML costs for categorical targets (Experiment 2). Although this result may be based at least partly on the specific manipulation, it is also in line with the multiprocess theory (McDaniel and Einstein, 2000; McDaniel et al., 2004; Einstein et al., 2005). According to this theory specific targets increase the involvement of automatic processing in prospective remembering, rendering performance less susceptible to load effects. Therefore, PL may only affect performance in prospective memory tasks that are likely to rely on strategic processes. Features of prospective memory tasks that enable processing transfer might limit the effect of PL (cf. Maylor, 1996; Meier and Graf, 2000). Our experiments were not designed to specifically investigate the interaction of processing overlaps and load effects. However, the degree of processing overlap between the requirements for the ongoing lexical decision task and the requirements for recognizing the prospective memory targets was rather moderate in the present study. Hence, features of prospective memory tasks which enable a higher degree of transfer appropriate processing might completely compensate the effects of PL.

In contrast to PL, RL mainly affected retrieval of the retrospective component. As post-experimental interviews indicated failure to retrieve the retrospective component is not due to forgetting the intention. Rather, it is due to a lack of processing resources to retrieve the planned action when the ongoing task must be disengaged. In everyday-life, it is common that different prospective memory targets are associated with different actions and retrospective memory load may often be high. Consequently, prospective memory performance can be affected by failure to retrieve the retrospective component, particularly during ongoing activities that require quick responses. However, it should be noted that in everyday-life prospective memory targets are often related to the content of the intended actions. Based on studies examining the association between prospective memory targets and intended actions the effect of RL may be limited to actions which are *not* closely related to the respective prospective memory target (Cohen et al., 2001; McDaniel et al., 2004; Meier et al., 2006a).

Significant PML costs (i.e., higher reaction times for the lexical decision task in the ongoing task compared to baseline) were found for both specific and categorical targets. This indicates that, in both experiments, processing resources were required for the prospective memory task. Therefore, this result is consistent with the PAM theory (Smith, 2003; Smith and Bayen, 2006). However, as Scullin et al. (2013) demonstrated, the engagement in monitoring depends on the expectations of the occurrence of prospective memory targets. Specifically, according to the dynamic multiprocess framework, monitoring is engaged in contexts in which targets are expected, disengaged in contexts in which it is not expected, and in the latter a probabilistic spontaneous retrieval mechanism can support prospective remembering (Scullin et al., 2013). It is a question for future research to determine how spontaneous retrieval may interact with different degrees of PL and RL.

In the present study, we varied the pace of the ongoing task to manipulate OTL. This manipulation affected the retrospective component, most likely it resulted in an unsolved competition for processing resources between processes required for disengaging from the ongoing task and processes required for retrieving the retrospective component. This result suggests that depending on the strength of the manipulation of OTL and depending on RL, OTL can either lead to a deficit in noticing the prospective targets (e.g., Marsh and Hicks, 1998) or a deficit in retrieval of the retrospective component (Experiment 2 of the present study). Depending on the demands of OTL it is also possible that it does not affect prospective memory performance at all (Otani et al., 1997; Marsh and Hicks, 1998). However, under some conditions OTL may also affect both components. Moreover, it is possible that both RL and OTL impact the division of resources between the ongoing task and the retrieval of the RM component. Thus, RL may also result in slower disengagement because fewer resources are directed to the ongoing task. Future research is necessary to distinguish between these possibilities.

To summarize, in the present study we systematically distinguished between different types of load that can occur in prospective memory situations. The distinction between OTL and PL is derived from the dual task requirements that are present in prospective memory tests. The distinction between PL and RL results from the two-component nature of a prospective memory task. We presented evidence that these types of load affect prospective memory performance differently. However, we believe that depending on the specific task requirements these effects can be moderated by other variables, for example by the degree of processing overlap between study and test phase processing, processing overlap between ongoing task and prospective memory task requirements and the association between prospective memory targets and the content of an intention.

## Conflict of Interest Statement

The authors declare that the research was conducted in the absence of any commercial or financial relationships that could be construed as a potential conflict of interest.

## Footnotes

^1^www.pstnet.com

^2^In an alternative analysis, the responses were scored correct for the groups with high RL, when the response corresponded to the absolute position of the prospective memory target in the ongoing task. A two-factorial ANOVA with PL and RL load as between-subject factors revealed a significant main effect of RL, *F*_(1,50)_ = 18.65, *p* < 0.01, *MSe* = 0.08; *η* = 0.27, (*M* = 0.97 for low RL and *M* = 0.63 for high RL). No other effect was significant (all *F*s < 0.1, all *p*s > 0.40).

^3^In an alternative analysis, the responses were scored correct for the groups with high RL, when the response corresponded to the absolute position of the prospective memory target in the ongoing task. A three-factorial ANOVA with PL, RL and OTL as between-subject factors revealed a significant main effect of RL, *F*_(1,111)_ = 31.46, *p* < 0.01, *MSe* = 0.10, *η* = 0.22 (*M* = 0.95 for low RL and *M* = 0.63 for high RL). No other effect was significant (all *F*s < 1.4, all *p*s > 0.24).
